# Chromatin remodeling dysfunction extends the etiological spectrum of schizophrenia: a case report

**DOI:** 10.1186/s12881-019-0946-0

**Published:** 2020-01-08

**Authors:** Alice Poisson, Nicolas Chatron, Audrey Labalme, Pierre Fourneret, Dorothée Ville, Marie Laure Mathieu, Damien Sanlaville, Caroline Demily, Gaëtan Lesca

**Affiliations:** 1 GénoPsy, Reference Center for Diagnosis and Management of Genetic Psychiatric Disorders, Centre Hospitalier le Vinatier and EDR-Psy Q19 Team (Centre National de la Recherche Scientifique & Lyon 1 Claude Bernard University), le Vinatier, 69500 Bron, CH France; 2 Institut Neuromyogène, métabolisme énergétique et développement durable, CNRS UMR 5310, INSERM U1217, Université de Lyon, Université Claude Bernard Lyon 1, Lyon, France; 3 Service de psychopathologie du développement, hôpital Femme-Mère-Enfant, hospices civils de Lyon, 69677 Bron cedex, France; 4 Institut des sciences cognitives CNRS UMR, 530467 boulevard Pinel, 69675 Bron cedex, France; 5 Faculté de médecine Lyon-Est, université Claude-Bernard - Lyon 1, 69003 Lyon, France; 6 Département de Neurologie Pédiatrique et Centre de Référence des Epilepsies Rares, Hôpital Femme Mère Enfant, Hospices Civils de Lyon, Centre Hospitalier Universitaire de Lyon, Lyon, France; 7 Neuropaediatrics Department, Femme Mère Enfant Hospital, Lyon, France; 8 Claude Bernard Lyon 1 University, Lyon, France

**Keywords:** Schizophrenia, Childhood onset schizophrenia, *CHD2*, Genetic counselling, Chromatin, Chromodomain helicase DNA-binding

## Abstract

**Background:**

The role of deleterious copy number variations in schizophrenia is well established while data regarding pathogenic variations remain scarce. We report for the first time a case of schizophrenia in a child with a pathogenic mutation of the *chromodomain helicase DNA binding protein 2 (CHD2)* gene.

**Case presentation:**

The proband was the second child of unrelated parents. Anxiety and sleep disorders appeared at the age of 10 months. He presented febrile seizures and, at the age of 8, two generalized tonic-clonic seizures. At the age of 10, emotional withdrawal emerged, along with a flat affect, disorganization and paranoid ideation, without seizures. He began to talk and giggle with self. Eventually, the patient presented daily auditory and visual hallucinations. The diagnosis of childhood onset schizophrenia (DSM V) was then evoked. Brain imaging was unremarkable. Wakefulness electroencephalography showed a normal background and some bilateral spike-wave discharges that did not explain the psychosis features. A comparative genomic hybridization array (180 K, Agilent, Santa Clara, CA, USA) revealed an 867-kb 16p13.3 duplication, interpreted as a variant of unknown significance confirmed by a quantitative PCR that also showed its maternal inheritance. Risperidone (1,5 mg per day), led to clinical improvement. At the age of 11, an explosive relapse of epilepsy occurred with daily seizures of various types. The sequencing of a panel for monogenic epileptic disorders and Sanger sequencing revealed a de novo pathogenic heterozygous transition in *CHD2* (NM_001271.3: c.4003G > T).

**Conclusions:**

This case underlines that schizophrenia may be, sometimes, underpinned by a Mendelian disease. It addresses the question of systematic genetic investigations in the presence of warning signs such as a childhood onset of the schizophrenia or a resistant epilepsy. It points that, in the absence of pathogenic copy number variation, the investigations should also include a search for pathogenic variations, which means that some of the patients with schizophrenia should benefit from Next Generation Sequencing tools. Last but not least, *CHD2* encodes a member of the chromodomain helicase DNA-binding (CHD) family involved in chromatin remodeling. This observation adds schizophrenia to the phenotypic spectrum of chromodomain remodeling disorders, which may lead to innovative therapeutic approaches.

## Background

The growing access to comparative genomic hybridization arrays has allowed for better identification of schizophrenia cases associated with the presence of deleterious copy number variations [1–5]. However, data regarding schizophrenia linked to pathogenic variations remain scarce, despite an increasing availability of diagnostic techniques. There is a dire need to clarify the benefit and modality of high-throughput genetic investigations in schizophrenia. This step is crucial to provide accurate genetic counseling, to further understand the pathological mechanisms underlying the disease and to imagine new innovative therapies. We describe here for the first time a case of childhood onset schizophrenia eventually linked to the presence of a pathogenic variant of *CHD2*, which encodes a chromodomain protein involved in neurogenesis, chromatin remodeling and gene expression.

## Case presentation

**Fig. 1:**
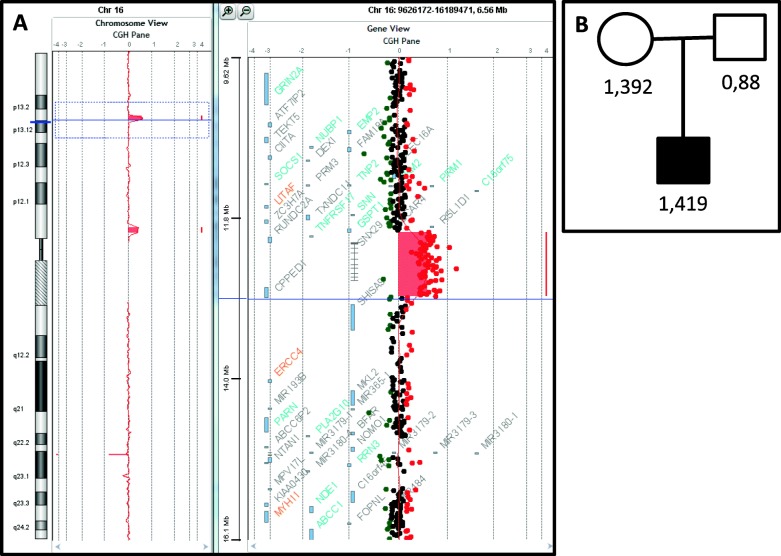
**a** Detail of the patient’s CGH array analysis showing a 16p13.3 interstitial duplication (867-kb). **b** qPCR ratios confirmed the duplication and showed its maternal inheritance

**Fig. 2:**
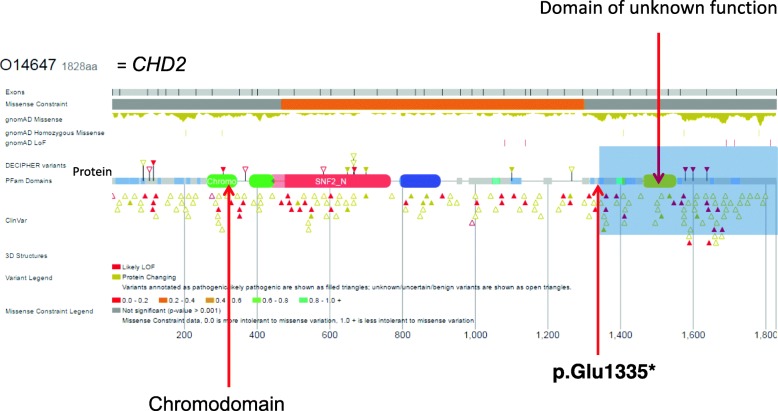
Representation of *CHD2* variant adapted from the DECIPHER browser. The heterozygous transition in *CHD2* (NM_001271.3: c.4003G > T) is predicted to produce a premature stop codon, p.(Glu1335*), which likely brings on non-mediated decay (NMD) so that no protein could be produced

**Fig. 3:**
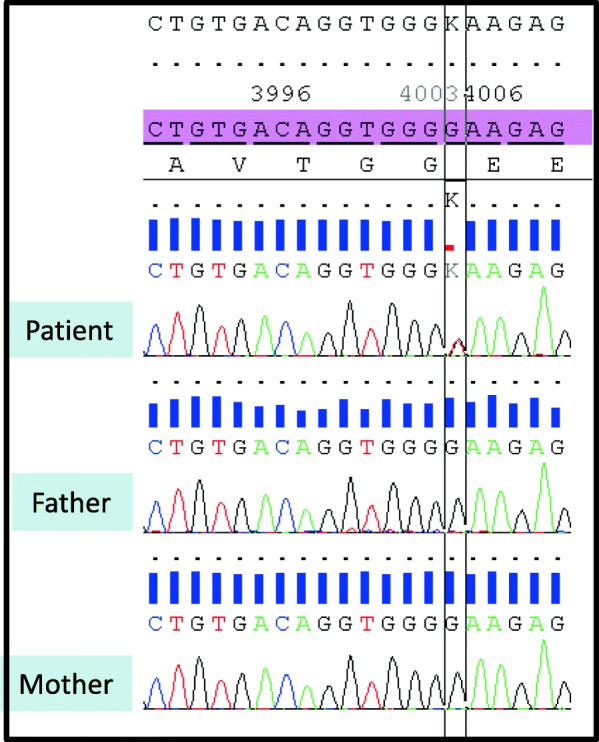
*CHD2* variant validated by Sanger sequencing. The variant (c.4003G > T) was found in the patient but was absent in both parents

The proband was the second child of unrelated parents with no remarkable medical history. The paternal grandfather had a history of simple, uncomplicated febrile seizures in infancy. The pregnancy and delivery were unremarkable, and the patients’ neurodevelopmental milestones were in the normal range. However, the mother described the appearance of sleep disorders at the age of 10 months that increased the sleep onset latency and nocturnal awakenings and ceased with co-sleeping. Not until the age of 6 could he sleep alone. The patient began school at the age of 3, but separation anxiety was severe and interfered with his learning. He was also described as a solitary child without other signs suggestive of an autism spectrum disorder. Between 19 months and 6 years of age, he experienced 7 typical febrile seizures. At 7 years of age, a global cognitive assessment revealed a heterogeneous profile with verbal intellectual quotient (IQ) of 78, perceptive IQ of 71 and speed processing index of 59. These results should, however, be interpreted with caution given the level of anxiety. At the age of 8, sodium valproate (400 mg per day, blood concentration: 52.4 mg/l) was introduced because of the occurrence of two spontaneous generalized tonic-clonic seizures with generalized spike-wave discharges upon interictal electroencephalography recording. Sodium valproate led to full seizure control. At the age of 9, anxiety escalation was noted, with death worries, iterative questioning, restlessness and panic attacks. At the age of 10, emotional withdrawal emerged, along with a flat affect, suicidal thoughts, irrelevant talk, delusions and paranoid ideation, with no recurrence of seizures. Confusion and disorganization were also noted. He began to talk and giggle with self. Eventually, the occurrence of daily auditory and visual hallucinations led to the patient’s hospitalization. The diagnosis of childhood onset schizophrenia (DSM V) was then evoked. This uncommon and severe phenotype associated with visual hallucinations led to further investigations. Brain magnetic resonance imaging was unremarkable. Wakefulness electroencephalography showed a normal background and some bilateral spike-wave discharges that did not explain the psychosis features and lacked contemporary clinical manifestation. Ammonemia, urinary organic acids, plasmatic amino acids, exchangeable copper, lysosphingolipids and oxysterols were in the normal ranges. A comparative genomic hybridization array (180 K, Agilent, Santa Clara, CA, USA) and quantitative polymerase chain reaction revealed an 867-kb 16p13.3 duplication inherited from the patient’s mother, which was interpreted as a variant of unknown significance (arr [hg19] 16p13.13p13.12(12,008,151-12,875,277) × 3 mat). (Fig. 1) The patient was given risperidone (1,5 mg per day), which led to a clinical improvement allowing hospital discharge. At the age of 11, an explosive relapse of epilepsy occurred with daily seizures of various types: absences, myoclonic fits, tonic and tonicoclonic seizures. No contemporary increase in behavioral disorders was noted. An electroencephalogram disclosed slow background activity, with bilateral diffuse spikes and waves predominating in the frontal area. Several tonic seizures were recorded. A photoparoxysmal response was recorded on electroencephalogram with photic stimulation. Progressive but significant improvement of epilepsy was obtained with lamotrigine (250 mg per day) in addition to valproic acid (1200 mg per day, blood concentration: 63.6 mg/l). The epilepsy phenotype, close to Lennox-Gastaut syndrome, led to the sequencing of a 93-gene panel for monogenic epileptic disorders. Capture was performed with the SeqCap EZ kit (Roche, Madison, WI, USA) and sequencing on a NextSeq500 (Illumina, San Diego, CA, USA). Gene panel sequencing disclosed the presence of a heterozygous transition in *CHD2* (NM_001271.3: c.4003G > T) that was predicted to produce a premature stop codon, p.(Glu1335*). (Fig. 2) This transition had not been reported previously in patients or in database of control individuals (gnomAD). Sanger sequencing from the patient and his parents confirmed the presence of the variant and indicated its de novo occurrence. (Fig. 3) The present truncating variation likely brought on non-mediated decay (NMD) so that no protein could be produced.

## Discussion and conclusion

De novo heterozygous truncating or missense variations of *CHD2* have been described in children with childhood-onset epileptic encephalopathy (OMIM # 615369) [6–9]. The growing access to genomic testing begets the continuous discovery of new, moderate phenotypes related to rare monogenic variants that were only associated at first with severe clinical pictures and variable expressivity of pathogenic rare variants is particularly well known in neuropsychiatric disorders [10] In that vein, pathogenic single-nucleotide variations or deletions of *CHD2* have also been linked to intellectual deficiency and behavioral disorders, including autism spectrum disorders, attention-deficit hyperactivity disorder, and challenging behaviors [6, 11–20]. Bernardo et al., described the occurrence of severe behavioral disorders and aberrant emotional and affective complex aspects disrupting social functioning in a 27 years old woman with a *CHD2* mutation [20]. Veroeven et al. reported on short-lasting psychotic episodes eventually linked to an untreated absence epilepsy as it disappeared concomitantly with the introduction of valproic acid [12]. Thomas et al. (2015) described the occurrence of schizophrenia in a 29-year-old patient with *CHD2* mutation and myoclonic seizures during infancy [16]. (Table 1) However, given the prevalence of schizophrenia during adulthood, no definitive link between *CHD2* and schizophrenia could be established at that time. In the present case, schizophrenia had a childhood onset, inaugurated the clinical degradation and was not improved by valproic acid. Seizures were well controlled during psychosis onset, and in particular, no myoclonic fit was observed during this period. Furthermore, when seizures reoccurred, psychosis was still well controlled. Overall, these findings suggest that schizophrenic disorders likely belong to the clinical spectrum of *CHD2* haploinsufficiency, as a distinct phenotype in association with epilepsy. This observation is in line with previous genome-wide association studies and rare variants studies that applied common pathways between schizophrenia, epilepsy, and intellectual deficiency [21–23]. To our knowledge, the 16p13.3 duplication affects genes that have not been related to neurodevelopment. However, we cannot exclude a modulating role of this copy number variation on the phenotype, as has previously been described for double-hit carrier patients [24, 25].

**Table 1:** Cases of *CHD2* mutations with behavioral disorders: main psychiatric and cognitive characteristics

Reference	Number of patients	Gender M/F	Mean Age (years)	Behavioral features (Number)	Cognitive profile	Brain MRI
[5]	6	4/6	14,5	ASD (2)	Moderate ID (2) Severe ID (4)	NA
[11]	4	NA	NA	ASD (4)	Cognitive impairment (4)	NA
[14]	9 (+ 1deletion)	6/4	17,9	ASD (4) ADHD (1) Challenging behavior (8) Psychotic features (1)	Mild to severe (10)	Progressive atrophy (3) Corpus callosum atrophy (3) Normal during adulthood (2)
[15]	2	2/0	NA	ASD	Mild d ID (1/2)	NA
[16]	1	1/0	26	Hyperactivit Autistic features	Severe ID	Normal
[17]	2	2/0		ASD Hyperactivity Developpemental delay Bruxism Stereotypic movements		NA
[18]	1	0/1	27	Limited social skills Psychotic disorder	Moderate ID	NA

*CHD2* encodes the chromatin organization modifier (chromodomain) helicase DNA-binding protein2 and a SNF2-related helicase/ATPase domain, involved in neurogenesis and chromatin organization [6, 26–29]. Chromodomains are highly evolutionary conserved among eukaryotes and interact with DNA conformation [30, 31]. Converging evidence has suggested a link between schizophrenia and epigenetic processes [32, 33]. However, most of our knowledge on this subject comes from in vivo animal studies, which are unable to reproduce the complexity of human mental disorders. In a murine model, CHD2 prevents suppressive chromatin formation in neurodevelopmentally regulated genes and allows the maintenance of embryonic stem cells in an undifferentiated state. It also remodels chromatin into a permissive state [34] via the replacement of histone H3 with histone H3.3 [29]. In human embryonic stem cells, *CHD2* haploinsufficiency seems to impair cortical interneurons specification, which may in turn lead to the electrophysiological dysfunction of these interneurons [26]. The impairment of the local inhibition usually provided by these interneurons may be involved in the occurrence of epilepsy and schizophrenia in children with *CHD2* haploinsufficiency. Interestingly Lamar suggests that, in a near future previously developed cancer drug such as Histone Deacetylase (HDAC) Inhibitors may be useful in neurological disease linked to *CHD2* dysfunction [29]. Given the data from this case report, schizophrenia might also benefit from such breakthrough in drugs development. Last but not least, to allow for better identification of schizophrenia cases associated with rare genetic disorders, the presence of warning signs (‘red flags’) would be greatly helpful [32, 33]. Previous data suggested that intolerance and resistance to psychotropic medication could be used as such red flags [35–37]. Based on the present case, we propose that schizophrenia onset before 13 years of age, visual hallucinations and resistant epilepsy should lead to genetic investigations. The role of pathogenic copy number variations in schizophrenia is now well established. We suggest that an exhaustive etiological investigation of patients with childhood onset schizophrenia, especially in the presence of other ‘red flags’ and in the absence of pathogenic copy number variation, should seek for intragenic pathogenic variants.

In conclusion, this case brings new elements in favor of a link between *CHD2* and schizophrenia. It highlights that schizophrenia may be, in some cases, a Mendelian disorder. We propose that a childhood onset of schizophrenia should be considered as a red flag and should lead to systematic genetic investigations. This case also underlines that, in the absence of pathogenic copy number variations, the investigations should also include a search for pathogenic variations, which means that some patients with schizophrenia should benefit from Next Generation Sequencing tools. Last but not least, *CHD2* encodes a member of the chromodomain helicase DNA-binding (CHD) family involved in chromatin remodeling. Thus, this observation adds schizophrenia to the phenotypic spectrum of chromodomain remodeling disorders, which may lead to innovative therapeutic approaches.

## Abbreviations

CHD: Chromodomain helicase DNA-binding
*CHD2*: *Chromodomain helicase DNA-binding 2*
IQ: Intellectual quotient
NMD: Non-mediated decay
